# Adolescents’ Behaviors as Moderators for the Link between Parental Self-Efficacy and Parenting Practices

**DOI:** 10.1007/s10826-016-0623-2

**Published:** 2016-11-22

**Authors:** Terese Glatz, Allison Cotter, Christy M. Buchanan

**Affiliations:** 1 Center for Developmental Research at JPS, Örebro University, Fakultetsgatan 1, Örebro, 701 82 SE Sweden; 2 Department of Psychology, Auburn University, 226 Thach Hall, Auburn, AL 36849 USA; 3 Department of Psychology, Wake Forest University, 415 Greene Hall, P.O. Box 7778 Reynolda Station, Winston-Salem, NC 27109 USA

**Keywords:** Family processes, Systems theory, Parenting practices, Adolescents’ externalizing behaviors

## Abstract

Based on theory that parents with higher levels of self-efficacy (PSE) should find it easier to parent effectively in the face of challenging child behaviors than should parents with lower levels of PSE, this study examines the link between PSE and parenting using children’s behaviors as potential moderators. Participants were 130 parents who had an older adolescent (*M*_age_ = 17.58) in addition to the target adolescent (*M*_age_ = 11.79), and both adolescents’ externalizing behaviors were used as moderators for the link between PSE and parenting of the target adolescent. Path analysis in Mplus showed that higher PSE was linked to more promotive parenting but only among parents who had an older adolescent with lower levels of externalizing behaviors. Among parents of adolescents with higher levels of externalizing behaviors, whose promotive parenting was significantly lower than other parents overall, PSE did not predict promotive parenting. The link between PSE and parenting did not differ depending on the target adolescents’ behavior. Findings suggest that the link between parents’ beliefs and parenting depends on the broader family context. More specifically, how PSE is linked to parenting practices depends at least partly on the experiences that parents bring from parenting an older adolescent to their interactions with a later-born adolescent. From a clinical perspective, parents might need guidance in how to think about their earlier parenting experiences when parenting a younger adolescent.

## Introduction

Parental self-efficacy (PSE) describes parents’ beliefs about being able to influence their child in a way that fosters his or her positive development and adjustment (Bandura 1977, 1997). Parents who believe that they are capable of influencing their children in a positive way are more likely to support their children’s skills, talents, and interests as well as acting in ways to prevent negative child adjustment (i.e., promotive parenting practices, Furstenberg et al. 1999) than are parents who do not believe they are capable of such influence (e.g., Ardelt and Eccles 2001; de Haan et al. 2009; Dumka et al. 2010; Glatz and Buchanan 2015a; Slagt et al. 2012). Despite this well-documented general association between PSE and promotive parenting, theoretically, how a person’s self-efficacy relates to his or her actions can differ as a function of the person’s context (Bandura 2002). Yet few studies have examined possible contextual moderators for the link between PSE and parenting.

When moderators of the link between PSE and parenting practices have been examined, results have been consistent with Bandura’s suggestion (Bandura 2002), showing that ecological or demographic factors moderate this association. For example, PSE has been found to be more strongly linked to promotive parenting practices among mothers than among fathers (Glatz and Buchanan 2015a) and among African American mothers than among European American mothers (Ardelt and Eccles 2001; Elder et al. 1995). Additionally, higher levels of PSE have been shown to predict more positive parent-child interactions especially when parents are also sensitive to the child’s developmental needs (e.g., Conrad et al. 1992; Wilson et al. 2014). Thus, previous research demonstrates that higher levels of PSE are indeed linked to more promotive parenting practices but that this link can depend on other factors, including parents’ ethnicity, gender, and sensitivity to the child’s developmental needs.

The type of child behaviors that parents are faced with might also influence the link between PSE and parenting. In fact, it has been argued that parents with higher levels of PSE should find it easier to parent effectively in the face of difficult and challenging child behaviors than should parents with lower levels of PSE (Jones and Prinz 2005). Conversely, when parents are facing easy-to-handle or more positive child behaviors, their level of PSE might not be a strong predictor of their subsequent parenting practices. This might be because positive child behaviors are likely to illicit positive parenting practices in general, regardless of parents’ level of PSE. Hence, the combination of PSE and a child’s behavior might interact in predicting parenting practices. Specifically, PSE should be more important for the level of positive parenting when a child exhibits higher levels of difficult behaviors than when the child exhibits less difficult behavior.

Another potential moderator for the link between PSE and parenting practices is parents’ experiences with other children that they have previously parented. Such a moderation would be consistent with a family systems perspective (e.g., Minuchin 1974), in which sub-systems within the family influence one another. In general, parents’ interactions with their children are often different (Whiteman et al. 2003). For example, parents tend to give more autonomy to, show more warmth to, spend more time with, and have fewer conflicts with their later-born child than with their first-born child (Lam et al. 2012; Shanahan et al. 2007a, b; Wray-Lake et al. 2010), at least during certain developmental periods. Additionally, according to research, the presence of multiple children, and even more so the type of experiences that parents have with those different children, is of importance for their parenting practices (Glatz and Stattin 2013; Whiteman and Buchanan 2002). Specifically, negative experiences with a first-born child are sometimes transferred into negative expectations and feelings when parenting their later-born child (Glatz and Stattin 2013; Whiteman and Buchanan 2002). Hence, parents’ earlier experiences seem to matter for their parenting practices of a later-born child. Whether parents’ experiences with an older child moderate the link between PSE and parenting of a younger child has not been examined empirically.

Parenting practices are also shaped to some extent by similarities and differences in children’s behaviors. In two previous studies, parents’ experience with an older child was shown to have an impact on parenting of the target child especially when the target child expressed behaviors similar to the older child (Glatz and Stattin 2013; Whiteman and Buchanan 2002). These results suggest that parenting practices might spill over from the parenting of one child to the parenting of another child particularly when children express similar behaviors. Hence, it is possible that when an older and younger child express similar behaviors, the primary predictor of parenting is the shared behavior and the parent’s level of PSE is less predictive of parenting.

By contrast, when parents experience different behaviors in their children, their parenting practices might depend on both the children’s behavior as well as their level of PSE. For example, parents often have more positive interactions with a child who expresses lower levels of externalizing behaviors compared to a sibling who exhibits more externalizing behaviors (Lam et al. 2012; Meunier et al. 2012), demonstrating that parents might use different parenting practices with two children who express different behaviors. In such situations, the level of PSE might become more influential for parenting practices, particularly with a younger child, given that parents with less experience might feel more uncertainty when handling these different child behaviors. This might be true especially when the younger child expresses more difficult behaviors than the older child as this situation presents parents with new parenting challenges, making their beliefs about their ability to influence the younger child potentially more crucial in determining their parenting actions. Hence, the level of PSE might predict the level of promotive parenting especially when parents have experienced relatively little difficult behavior in an older child and now face more difficult behavior in a subsequent child.

In this study, participants were parents with two adolescent children, and we examined whether parents’ perceptions of difficult adolescent behaviors moderated the link between PSE and parenting of the younger adolescent, identified as the “target” adolescent. We focused on early adolescence, as this is a time when PSE is at especially low levels (Ballenski and Cook 1982; Glatz and Buchanan 2015b), which have been shown to have negative consequences for parenting practices among parents of children in this age range (Glatz and Buchanan 2015a). We expected that higher levels of PSE would predict more promotive parenting especially when parents face more difficult behaviors in the target adolescent. In contrast, we expected that when parents face less difficult behaviors in the target adolescent, the level of promotive parenting would be relatively high and independent of the level of PSE.

Concerning the impact of an older adolescent’s behavior on the link between PSE and parenting of the target adolescent, we posed two plausible hypotheses: (1) higher levels of PSE should be linked to more promotive parenting only when parents have experienced difficult behaviors in their older adolescent, or (2) among parents who experienced difficult behaviors in their older adolescent, such negative experiences would be transferred into less promotive parenting practices of the target adolescent, regardless of their level of PSE. We also had hypotheses concerning the combination of both adolescents’ behavior. We expected that among parents who experienced less difficult behavior in their older adolescent and more difficult behaviors in the target adolescent, higher levels of PSE would be linked to higher levels of promotive parenting. On the contrary, PSE should not be a strong predictor of parenting when parents faced more difficult behaviors in the older adolescent than in the target adolescent or when parents faced similar behaviors in both of their adolescents.

## Method

### Participants

The sample was drawn from a longitudinal project involving 398 parents (284 mothers and 114 fathers) of a target adolescent child in sixth or seventh grade. We used reports from a sample of 130 parents (89 mothers and 41 fathers) from the first time point of the data collection in 1999–2000. These parents were eligible for the current study because they had an older adolescent (*M*_age_ = 17.58, SD = 3.19) in addition to the target adolescent (*M*_age_ = 11.79, SD = .66), and they reported on all study variables.

The majority (72 %) of the sibling pairs were within three to six years of one another in age (*M*_age difference_ = 5.78 years; SD = 3.29); 55 % of the sibling pairs were of the same sex and 44 % were of the opposite sex. Concerning parents’ ethnicity, 67 % were European American, 32 % were African American, and 1 % was Hispanic. This ethnic distribution is similar to the current national and state ethnic breakdown (62 and 64 % White, Non-Hispanic; 13and 22 % African American; and 18 and 9 % Hispanic, U.S. Census Bureau 2010–2015). Family income was distributed as follows: Over US$150,000 (2 %), $75,000–$150,000 (28 %), $75,000–$40,000 (43 %), $40,000–$20,000 (19 %), and less than $20,000 (8 %). Median income was $75,000–$40,000—a range that includes both current national and state median income ($53,482 and $46,693, respectively; U.S. Census Bureau 2010–2015). Concerning highest level of parental education, 2 % had less than a high school degree, 18 % had a high school degree, 48 % had some college or vocational school, 25 % had a college degree, and 7 % had a graduate or professional degree. The majority (72 %) of parents were married to the target adolescents’ other biological parent.

### Procedure

Parents were recruited through two public middle schools located in the southeastern United States. Some of the parents came from the same families (48 %), whereas in other families, only the mother (48 %) or only the father (4 %) participated. In families with two participating parents, they were instructed to fill out the surveys separately. Once parents agreed to participate, they were mailed response scales that were used during a telephone interview, which lasted approximately one hour. Each parent was compensated $50 for his or her participation in the project.

### Measures

#### Parental self-efficacy for the target adolescent

Parents completed a five-item scale of parental self-efficacy (Freedman-Doan et al. 1993), focusing specifically on parents’ perceived influence on the target adolescents’ free-time activities and school adjustment. This measure has been used in previous studies and has shown to predict parenting practices (Glatz and Buchanan 2015a, b). Parents rated how much they thought they could influence the target adolescent, and the following are example items: “To get the child to stay out of trouble in school,” and “To prevent the child from doing things they do not want him or her to do outside the home.” Parents responded on a Likert scale ranging from 1 (*Very little*) to 7 (*A great deal*). Cronbach’s alpha for this scale was .80.

#### The target adolescents’ externalizing behaviors

Parents’ perceptions of the target adolescents’ externalizing behaviors were measured with the Child Behavior Checklist (CBCL; Achenbach 1991). This scale included 33 descriptions of difficult behaviors; examples are “Disobedient at home,” “Gets in many fights,” “Stubborn, sullen, or irritable,” and “Uses alcohol or drugs.” Response options ranged from 0 (*Not true [as far as I know]*) to 2 (*Very true or often true*). The CBCL has demonstrated strong associations with other measures of child externalizing behaviors as well as positive and negative parenting behaviors (e.g., Gallitto 2015; Pearl et al. 2014). Cronbach’s alpha for this scale was .81.

#### The older adolescents’ externalizing behaviors during early adolescence

We used the “risk-taking/rebellious” and “problem behaviors” subscales from Whiteman and Buchanan (2002) to measure parents’ experiences of externalizing behaviors in their older child when he or she was in early adolescence (12–14 years of age). Parents were asked to think about the closest aged older sibling to the target adolescent and respond to statements capturing this child’s risk-taking, rebelliousness, and problematic behaviors during the specific time period. We combined the 10 items from the two subscales into one composite scale in order to acquire an overall measure of externalizing behaviors; example items are “He/she is/was defiant,” “He/she was difficult to get along with,” and “He/she hung out with a crowd you disapprove/disapproved of.” Response options ranged from 1 (*Strongly agree*) to 7 (*Strongly disagree*). Cronbach’s alpha for this scale was .90.

#### Parenting of the target adolescent

Promotive parenting practices with the target adolescent were assessed using two subscales from the Alabama Parenting Questionnaire (APQ; Frick et al. 1999): The Positive Parenting subscale and the Parental Involvement subscale, which have been shown in previous studies to be linked to PSE among parents of adolescents as well as to adolescents' externalizing behaviors (Glatz and Buchanan 2015a; Gryczkowski et al. 2010). Six items were included in the Positive Parenting subscale; examples are “You compliment your child when he or she does something well,” and “You hug or kiss your child when he or she has done something well.” The Parental Involvement subscale consisted of 10 items; examples are “You have a friendly talk with your child,” and “You attend PTA meetings, parent teacher conferences, or other meetings at your child’s school.” For both subscales, parents responded to a scale ranging from 1 (*Never*)–5 (*Always*). A recent examination of the factor structure of the APQ among parents of adolescents ages 11–18 (Zlomke et al. 2014) suggested that these two subscales might be combined into a single measure. In our data, these two scales correlated moderately (.62, *p* < .001), and in order to use the same approach as the measurement of the older adolescent’s externalizing behaviors and to follow the suggestion by Zlomke et al. (2014), we decided to collapse the two scales into an overall measure of promotive parenting practices. Cronbach’s alpha for the collapsed scale was .90.

## Data Analyses

To examine main and interaction effects, we performed a path analysis with observed variables using Mplus 7.11 (Muthén and Muthén 1998–2012) with the maximun likelihood estimator. In this analysis, we used PSE, both adolescents’ externalizing behaviors, and four interaction terms as simultaneous predictors of parenting of the target adolescent. The interactions were computed using PSE for the target adolescent and parents’ perceptions of externalizing behaviors in the adolescents (Target adolescent externalizing*Older adolescent externalizing; PSE*Target adolescent externalizing; PSE*Older adolescent externalizing; and PSE*Target adolescent externalizing*Older adolescent externalizing). All variables were mean-centered before computing the interaction terms.

**Fig. 1:**
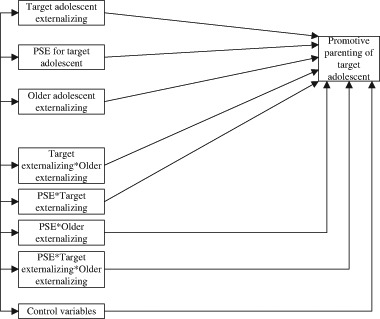
Analytical model. Control variables are: Whether one or two parents participated in the study and other significant demographic variables from the zero-order correlation analysis

In the path analysis, we also controlled for the impact of potentially important covariates. To decide what covariates to include, we first examined zero-order correlations between several demographic variables (parents’ ethnicity, both adolescents’ and parents’ sex and age, age difference between the siblings, family income, parents’ educational level, and parents’ marital status) on the one hand and promotive parenting practices on the other. Additionally, as noted earlier, in some families, both parents participated whereas in other families only one parent participated. To avoid potential biases in the results because of this inter-family dependency, we used this dichotomized variable (whether one or two parents from the same family participated in the project) as a covariate in the path analysis (together with other significant demographic variables from the zero-order correlation analysis). The analytical model is illustrated in Fig. 1.

To evaluate the model fit, three indices were used: the comparative fit index (CFI), the Tucker-Lewis index (TLI), and the root mean square error of approximation (RMSEA). CFI and TLI values above .90 and RMSEA values of .06 or lower are considered indicators of an acceptable fit between the hypothesized model and the observed data (Hu and Bentler 1999), so these values were used as cutoffs in this study.

## Results

Correlations, means, and standard deviations for all study variables are reported in Table 1. All study variables were correlated in the expected direction, with the target adolescents’ externalizing behavior being the strongest correlate of parenting practices. Three of the demographic variables from the zero-order correlation analysis correlated significantly with the parenting outcome variable: Whether one or two parents participated in the study, parents’ sex, and parents’ marital status (see Table 1).

**Table 1:** Correlations, means (*M*), and standard deviations (SD) for the study variables

Variable	1	2	3	4	5	6	7	*M*	SD
1. PSE	–							5.73	1.01
2. Promotive parenting	.26**	–						4.31	.47
3. Target adolescent externalizing	−.42***	−.29**	–					.30	.19
4. Older adolescent externalizing	−.35***	-.21*	.25**	–				2.90	1.39
5. Parents’ sex	.06	.26**	−.13	.00	–			–	–
6. One vs. two parents reporting	.06	.18*	−.09	−.05	.40***	–		–	–
7. Marital status	.01	−.22*	.00	.01	−.20*	−.50***		–	–

*Note*. Parents’ sex: 1 = fathers, 2 = mothers; one vs. two parents reporting: 1 = one parent, 2 = two parents; marital status: 1 = married to child’s other biological parent, 2 = not married to child’s other biological parent. *N* = 130

*p < .05; **p < .01; ***p < .001

The path analysis (see Fig. 1) showed a very good fit to the data, *χ*² = 18.78 (21), *p* = .599; RMSEA = .00; CFI = 1.00; TLI = 1.04. The results from this analysis are presented in Table 2. Concerning the control variables, parents’ marital status and sex were still significant predictors of parenting. Parents who were married to the target child’s other biological parent reported more promotive parenting practices than did parents who were not married to the other parent. Additionally, mothers reported more promotive parenting practices than did fathers. Despite being significantly correlated with promotive parenting in the zero-order correlation, whether one or two parents participated in the study was not a significant predictor in the path analysis. Concerning the main study variables, two out of three were significant predictors of promotive parenting: Higher levels of PSE and less externalizing behavior in the target adolescent were significantly related to more promotive parenting practices toward the target adolescent. The older adolescents’ externalizing behavior was not a significant predictor for parenting of the target adolescent.

**Table 2:** Results of the model examining the moderating effect of the older and the target adolescents’ externalizing behaviors on the link between PSE and parenting of the target adolescent

	Promotive parenting		
	*β*	SE	*p*
PSE for target adolescent	.20	.10	.039
Target adolescent externalizing behaviors	−.25	.10	.012
Older adolescent externalizing behaviors	−.15	.09	.080
Target externalizing*Older externalizing	−.09	.11	.426
PSE*Target externalizing	.09	.17	.608
PSE*Older externalizing	−.28	.10	.007
PSE*Target externalizing*Older externalizing	−.15	.18	.410
Parents’ sex	.17	.09	.046
One vs. two parents reporting	−.02	.10	.819
Marital status	−.19	.09	.033

*Note.* Parents’ sex: 1 = fathers, 2 = mothers; one vs. two parents reporting: 1 = one parent, 2 = two parents; marital status: 1 = married to child’s other biological parent, 2 = not married to child’s other biological parent. *N* = 130

**Fig. 2:**
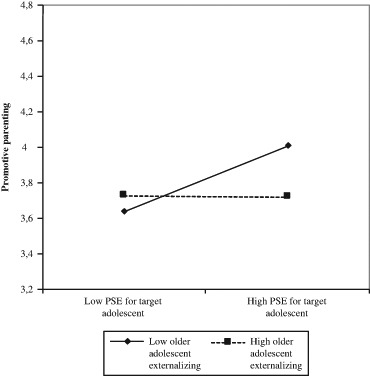
Interaction involving PSE for the target adolescent and the older adolescents’ externalizing behavior predicting promotive parenting of the target adolescent. *N* = 130

Of the interactions, the “PSE*Older adolescent externalizing” was the only significant predictor of promotive parenting. The interaction is depicted in Fig. 2. This graph demonstrates that higher levels of PSE were linked to higher levels of promotive parenting practices especially when parents reported lower levels of externalizing behaviors in their older adolescent. The slope set at one SD below the mean was significant (*B* = .15, SE = .05, *p* = .006), but the slope set at one SD above the mean was not significant (*B* = .04, SE = .05, *p* = .382).

## Discussion

In this study, we examined adolescents’ externalizing behavior as a moderator of the link between PSE and promotive parenting practices. Consistent with theory and previous research (e.g., Bandura 1977, 1997; Glatz and Buchanan 2015a; de Haan et al. 2009; Dumka et al. 2010; Slagt et al. 2012), higher PSE for the target adolescent child was linked to more promotive parenting practices of this particular child, but the strength of the association depended on the behavior of the older adolescent. The results support family systems theory and suggest that how PSE is linked to parenting practices can depend on dynamic processes taking place among and between family members. Specifically, parents’ interactions and experiences that take place in one sub-system of the family are important for their parenting choices within another sub-system as well as for how PSE is linked to these parenting choices.

The target adolescents’ externalizing behaviors were directly linked to promotive parenting practices, which is in line with traditional theories about child effects on parenting (e.g., Bell 1968; Maccoby and Martin 1983) and research (e.g., de Haan et al. 2013; Glatz and Buchanan 2015a; Glatz et al. 2011; Hafen and Laursen 2009; Kerr and Stattin 2003; Reitz et al. 2006; Slagt et al. 2012). In contrast to our hypothesis, however, in which we expected that the level of PSE would be linked to parenting of the target adolescent especially when the target adolescent showed higher levels of externalizing behaviors, the target adolescents’ behavior did not moderate the link between PSE and parenting. Rather, higher PSE was linked to more promotive parenting practices independent of the target adolescents’ behavior. This result is important and contrasts the theoretical idea that high levels of PSE allow parents to parent more effectively in the face of difficult child behaviors (Jones and Prinz 2005). The unexpected results of this study suggest that higher levels of PSE do not specifically buffer parents from acting negatively in face of difficult child behaviors; PSE is equally likely to promote more positive engagement of the sort assessed here across the spectrum of difficult behaviors. It should be mentioned, however, that only one type of child behavior among one age group (young adolescent) was examined. Other behaviors or samples of children in other ages (e.g., infants, toddlers, pre-school children) might offer different results. Additionally and importantly, the results of this study are based on one-time correlational data, and it is possible that children’s externalizing behavior would moderate the link between PSE and parenting over time. More research is needed using different samples and child behaviors as well as longitudinal data.

The second set of hypotheses involved the older adolescents’ behavior as a moderator for the link between PSE and parenting of the target adolescent. Although the target adolescent’s externalizing behavior did not moderate this link, the older adolescents’ externalizing behavior did. Of the two potential hypotheses we posed for this moderation, the significant interaction between the older adolescents’ externalizing behaviors and PSE in predicting promotive parenting of the target adolescent supported a spillover hypothesis. More specifically, parents whose older adolescent exhibited high levels of difficult behavior reported relatively low levels of promotive parenting practices with the target adolescent independent of their level of PSE. By contrast, among parents who experienced less difficult behavior in their older adolescent, levels of PSE were linked to levels of promotive parenting practices. Hence, this result suggests that parenting of a later-born child might be shaped by parents’ self-efficacy particularly when interactions have been enhanced by positive earlier parenting experiences. When parents report high levels of externalizing behaviors in their older adolescent, the link between PSE and parenting of a later-born adolescent was weakened, possibly because these previous negative parenting experiences create unduly negative expectations for the target adolescent, which outweighs the potential role of PSE.

Finally, in this study, we examined the potential moderating role of two adolescent children’s behaviors for the link between PSE and parenting. Hence, in addition to examining each as individual moderators, we examined the similarities and differences in the adolescents’ behaviors as a moderator. Based on earlier research, we hypothesized that among parents who reported more externalizing behaviors in the target adolescent than in their older adolescent, their level of PSE would matter more for their parenting than it would among parents who faced similar levels of difficult behaviors in their adolescents. We did not find support for this hypothesis, as the three-way interaction was non-significant. One explanation for this lack of finding concerns power. We had only 130 parent-child triads, and this sample size might have restricted the ability to capture true significance in the three-way interaction. Another explanation might lie in the outcome measure. We examined parenting practices rather than parental beliefs, which have been previously studied as an outcome of two children's behaviors (Glatz and Stattin 2013; Whiteman and Buchanan 2002). Parents’ experiences with two adolescents might be especially important for their beliefs (Weiner 1976) and might help form parents’ ideas about typical adolescent behaviors or beliefs about parenting ability (Glatz and Stattin 2013, Whiteman and Buchanan 2002). Experiences with two adolescents, however, might not be enough to change parenting behaviors. Parents’ behaviors, rather than their beliefs, might be more a reaction to the target adolescents’ behavior and their earlier experiences separately and not a combination of these. Studies using other parenting outcomes and adolescent behaviors should continue to examine the impact of similarity and dissimilarity in children’s behaviors for the link between PSE and parenting practices.

Limitations of this study include a somewhat small sample, which prevented us from performing more complex follow-up analyses. For example, we did not have enough power to examine differences in the analytical model between sibling pairs depending on their sex constellation (girl-girl, girl-boy, boy-girl, boy-boy), which might result in different parenting practices (e.g., Crouter et al. 1995; Shanahan et al. 2007a). Another potential follow-up analysis that was not performed in this study because of the small sample size is a more thorough examination of the sibling age gap in order to see whether the size of this gap affects the degree of spillover. Although these questions were not examined, we used strategies to increase the sample size in order to be able to perform the present analyses, such as including all available parent reports. Given that both parents participated in some families, some mothers and fathers in this study were reporting on the same child. Although this enabled the examination of the research questions and this dependency was controlled for in the analyses, this strategy can also be seen as a limitation of this study. In future studies, it will be important to include these additional controls and possible moderators, necessitating a larger sample of parents.

Another limitation with the present study is the use of parents as the sole reporter. Although the use of parent-reported measures was justified by our interest in parents’ perceptions of their children’s behaviors, using only parent-reported measures might increase the risk of one-reporter bias. However, similar associations among PSE, parent-reported adolescent behaviors, and parenting practices to those found in this study have been found in earlier studies using adolescents’ reports of their own behaviors (Glatz and Buchanan 2015a). This gives us more confidence in the results than we would have otherwise. Still, the results should be interpreted with the potential one-reporter bias in mind. Finally, because we used one-time correlational data, the findings do not inform about longitudinal processes or causal relations. Our interpretation that PSE predicts promotive parenting, although with different strength depending on a child’s behavior, is based on theory and prior research. It is, however, also possible that promotive parenting predicts PSE differently depending on a child’s behavior. Therefore, it will be important to explore possible processes in future studies with longitudinal data.

This study also had several strengths. It examined the idea that parents’ beliefs about their influence might be linked to their parenting practices differently depending on past and present parenting experiences. Of great importance is the examination of the theoretical assumption that parents are able to parent more effectively particularly in the face of difficult child behaviors if they believe in their ability to influence the child. The results of this study do not support this idea for either the target or older adolescent. Concerning the target adolescents’ behavior, the findings suggest that PSE is linked to promotive parenting practices independent of this child’s externalizing behaviors, supporting a main effect of PSE on parenting despite simultaneously examining the impact of difficult behaviors. On contrary, the older adolescent’s behavior moderated the link between PSE and parenting, supporting a spillover hypothesis. Further, in this study, we examined the type of experience (i.e., externalizing behaviors rather than only the presence of an older adolescent) that parents have with multiple children, as this has been suggested to be important (Glatz and Stattin 2013; Whiteman and Buchanan 2002). Feelings of mastery have been argued to be the most effective way to improve a person’s self-efficacy (Bandura 1977). This study showed that whether parents have mastered parenting their older adolescent (i.e., more or less externalizing behavior) was also important for the link between PSE and parenting practices for a younger child. In other words, our finding suggests that not only might feelings of mastery increase a person’s self-efficacy (Bandura 1977), but they might also influence the extent to which a person’s self-efficacy predicts his or her behavior.

This study brings together theory on PSE (Bandura 1977, 1997) and family systems (e.g., Minuchin 1974), enhancing our understanding about the circumstances under which high levels of PSE are linked to more promotive parenting practices. The results suggest that the link between PSE and parenting practices depends at least partly on the type of experiences that parents bring from parenting an older adolescent to their interactions with a later-born child. Based on this finding, it might be important that parenting programs address how parents interpret and use their earlier parenting experiences when parenting a younger adolescent. Particularly, it might be important to help parents reflect on negative experiences with an older child, as these types of experiences might, to some extent, outweigh the impact of PSE on their parenting practices of a later-born adolescent. For parents who have experienced high levels of externalizing behavior in their earlier-born child, it might be less effective to focus only on bolstering PSE. Instead, or in addition, it might be better to help these parents recognize that the parenting of their later-born children is a new experience and opportunity that can be different from their previous negative experiences with an earlier-born child.
